# Sexual Dysfunction in Cervix Cancer Survivors After Surgery and/or Radio(chemo)therapy: A Bayesian Network Analysis Based on the SENECA Study Cohort

**DOI:** 10.3390/cancers18162683

**Published:** 2026-08-19

**Authors:** Adriel Ngo, Kari Tanderup, Therese Juul, Gunn Ammitzbøll, Jakob Tanderup, Peter Christensen, Susanne O. Dalton, Lars U. Fokdal, Christoffer Johansen, Susanne K. Kjær, Monica Serban

**Affiliations:** 1 Department of Medical Biophysics, University of Toronto, Toronto, ON M5G 2M9, Canada; 2 Radiation Medicine Program, Princess Margaret Cancer Centre, Toronto, ON M5T 1P5, Canada; monica.serban@uhn.ca; 3 Danish Center for Particle Therapy, Aarhus University Hospital, 8200 N Aarhus, Denmark; karitand@rm.dk; 4 Department of Clinical Medicine, Aarhus University, 8000 C Aarhus, Denmark; therjuul@rm.dk (T.J.); petchris@rm.dk (P.C.); 5 Department of Surgery, Aarhus University Hospital, 8200 N Aarhus, Denmark; 6 Cancer Survivorship, Danish Cancer Institute, 2100 Copenhagen, Denmark; gunnam@cancer.dk (G.A.); sanne@cancer.dk (S.O.D.); 7 Danish Research Center for Equality in Cancer (COMPAS), Department of Clinical Oncology & Palliative Care, Zealand University Hospital, 4000 Roskilde, Denmark; 8 Department of Applied Mathematics and Computer Science, Technical University of Denmark, 2800 Kgs. Lyngby, Denmark; jaktan@dtu.dk; 9 Institute of Clinical Medicine, Faculty of Health Sciences, University of Copenhagen, 2400 Copenhagen, Denmark; 10 Department of Oncology, Aarhus University Hospital, 8200 Aarhus, Denmark; lars.fokdal@auh.rm.dk; 11 Department of Oncology, Copenhagen University Hospital, Rigshospitalet, 2100 Copenhagen, Denmark; christoffer.johansen@regionh.dk; 12 Virus, Lifestyle and Genes, Danish Cancer Institute, 2100 Copenhagen, Denmark; susanne@cancer.dk; 13 Department of Gynecology, Copenhagen University Hospital, Rigshospitalet, 2100 Copenhagen, Denmark; 14 Department of Radiation Oncology, University of Toronto, Toronto, ON M5G 2M9, Canada

**Keywords:** cancer survivorship, sexual functioning, cervix cancer, treatment side effects

## Abstract

Background: To better understand the complexity of sexual dysfunction, we aim to construct multi-factorial explanatory models of sexual dysfunction using data from participants enrolled in the Danish cross-sectional Study of latE effects for those liviNg through cErvical CAncer (SENECA). Methods: In a nationwide survey, patient-reported outcomes (PRO) were collected from 2002 cervical cancer survivors treated with surgery and/or radio(chemo)therapy between 2005–2020 and a reference cohort consisting of 7853 women without a history of cancer. PROs in the EORTC C30/CX24 questionnaires were analysed. Bayesian Network models were developed for sexual activity and dyspareunia in reference and cancer survivor cohorts, resulting in four explanatory models. The reference cohort served as a structural prior for the cancer models. Sexual activity models included participants with complete data (6706 reference, 1694 cancer), while dyspareunia models included those reporting sexual activity in the past four weeks (4521 reference, 996 cancer). Results: Respondents were stratified by treatment received: (1) reference group (*n* = 7853), (2) surgery alone (*n* = 1285), (3) radio(chemo)therapy (RCT) with/without prior surgery (*n* = 951). Sexual inactivity was reported by 28%, 29% and 49% of reference, surgery and RCT groups respectively, and dyspareunia by 23%, 39% and 54%. Sexual activity was associated with age, treatment modality and social functioning. Social functioning was associated with cognitive and role functioning, global QoL, and financial difficulties, all of which were associated strongly with fatigue. Dyspareunia was associated with vaginal functioning and soreness. The strong associations observed in the cancer survivor cohort between fatigue and functional outcomes/QoL/sexual activity were absent in the reference cohort. Conclusions: Sexual inactivity and dyspareunia were more common in cervical cancer patients, particularly in those treated with RCT compared with the reference cohort. Differences in explanatory factors between cancer and reference cohorts highlight the need for cancer-specific approaches to sexual rehabilitation.

## Simple Summary

Cervical cancer survivors are known to suffer from impaired sexual quality of life compared to reference populations. The aim of our study was to construct explanatory models of sexual dysfunction using data from the Danish cross-sectional Study of latE effects for those liviNg through cErvical CAncer (SENECA). Patient-reported outcomes from 2002 cervical cancer survivors and a reference cohort of 7853 women without history of cancer were analysed. Bayesian Network structure learning was performed, with the reference cohort models used as a structural prior for the cancer models. Sexual dysfunction was more prevalent in cervical cancer survivors compared to the reference population, especially those treated with RCT. Analysis of the BN models identified strong associations unique to the cancer cohort, particularly involving fatigue, demonstrating the need for cancer-specific, multidisciplinary rehabilitation strategies.

## 1. Introduction

Patients treated for cervical cancer are known to suffer from impaired sexual quality of life (QoL) compared to reference populations [1,2,3,4]. Generally, sexual health is an important aspect of QoL which may be influenced by physical, psychological, and social factors. The complex nature of sexual dysfunction highlights the need for a multi-factorial perspective to introduce sexual rehabilitation practice following successful cancer treatment [5,6].

Primary measures of sexual function include levels of sexual desire, arousal, orgasm, and dyspareunia, all of which may affect sexual activity [7]. Sexual dysfunction can negatively affect body image, self-esteem, and intimate relationships [8]. However, sexual dysfunction remains distinct from sexual inactivity as lack of sexual activity may be due to relationship factors or personal preference rather than impairment in sexual function.

Treatment of cervical cancer depends primarily on the FIGO (International Federation of Gynaecology and Obstetrics) stage [9] and may include surgery alone in limited stage disease, and radio(chemo)therapy (RCT) or chemotherapy alone in advanced stage disease. Both surgery and RCT are associated with a negative impact on sexual function [10].

Surgery may result in scarring formation and pelvic nerve damage, which may lead to vaginal stenosis, vaginal dryness, and bladder as well as bowel dysfunction [11,12]. Minimally invasive surgery has been observed to improve quality of life outcomes [1].

Radiotherapy (RT) is associated with damage of the vaginal mucosa, potentially leading to vaginal atrophy, vaginal shortening/stenosis, fistula, fibrosis, telangiectasia, mucositis, dryness, and scarring [13,14,15]. Furthermore, bladder and bowel dysfunction are common side effects of RT which may have an impact on daily activities and body image in cancer patients [16]. Chemotherapy can cause vaginal inflammation, dryness, irritation, and neuropathy [11]. RT causes premature menopause in premenopausal women.

Bayesian Networks (BNs) are probabilistic graphical models capable of learning complex multi-factorial relationships from data [17]. BNs may be designed as predictive or explanatory models. Predictive models use baseline data to predict future outcomes in a population; explanatory models aim to identify underlying relationships among variables which best explain observed data [18]. Explanatory modelling is used in this study to investigate associative pathways following treatment.

BN structures are represented by directed acyclic graphs comprised of nodes, representing variables in the data, and arcs, representing conditional independence relationships between variables. Each arc is directed, denoting parent and child relationships between nodes, and the graph is acyclic, prohibiting any set of arcs which would form a closed loop. BNs satisfy the condition that any node in the network, given its parent nodes, is conditionally independent of all other nodes in the network which are not connected, directly or indirectly, to its child nodes.

The aim of this study was to identify factors associated with sexual dysfunction by constructing multi-factorial explanatory models of sexual inactivity and dyspareunia among survivors of cervical cancer. We used data from participants enrolled in the Study of latE effects for those liviNg through cErvical CAncer (SENECA), a retrospective cross-sectional study designed to investigate late effects of cervical cancer treatment.

To the best of our knowledge, our study is the first application of BNs for explanatory modelling of sexual function in cervical cancer patients. Additionally, this paper proposes a novel methodology for use of a reference population as a prior distribution for BN modelling in cancer populations.

## 2. Materials and Methods

Patient-reported outcomes (PROs) were collected from consecutive women who were diagnosed with cervical cancer between 2005 and 2020, as well as a population-based reference cohort of women with no history of cancer who were identified from the Danish Civil Registration System (CRS). The reference cohort was selected of similar age distribution to the cervical cancer cohort.

**Figure 1:**
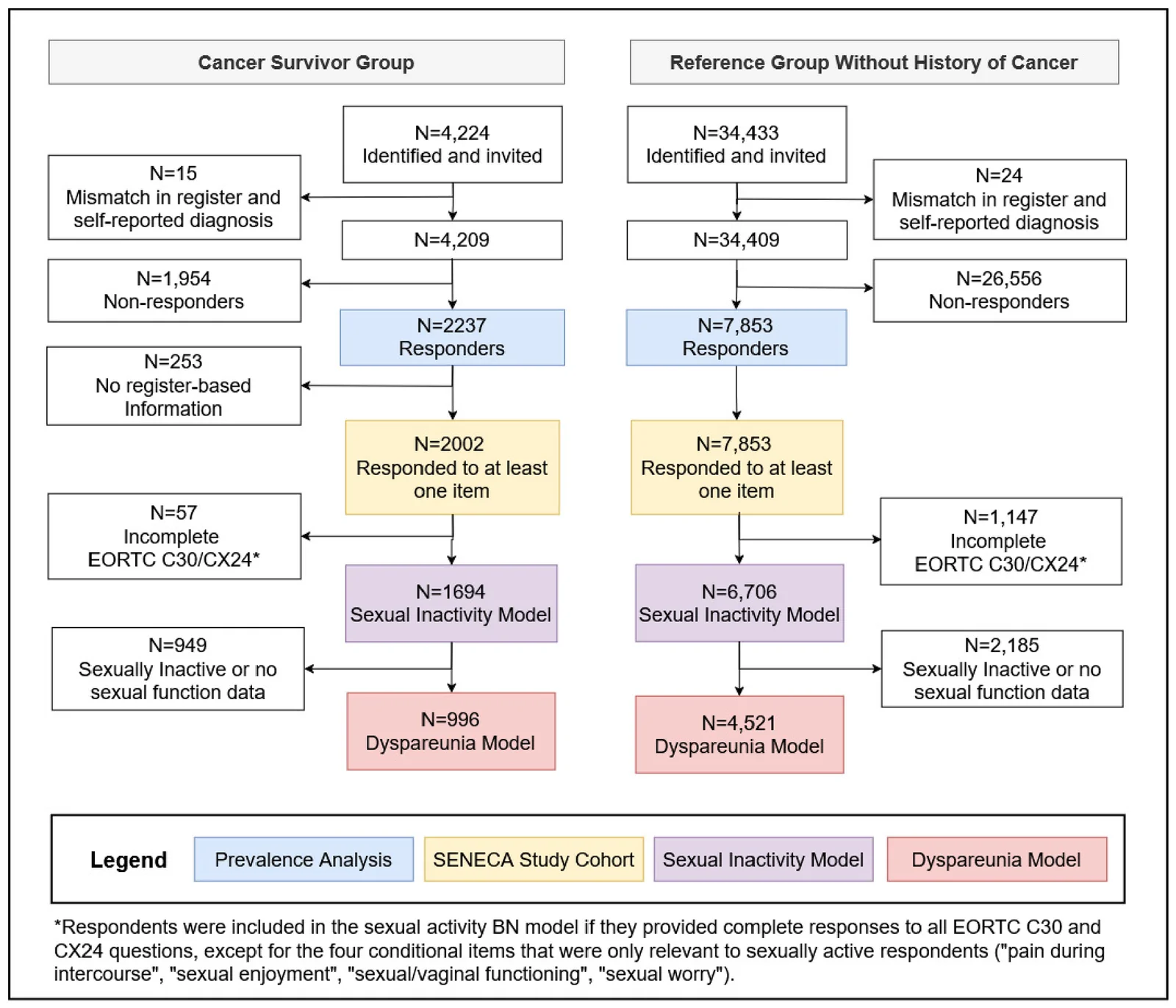
Participant inclusion flowchart for SENECA study cancer survivor group and reference group of women without history of cancer. Colours indicate the responder cohorts used for: prevalence analysis, the SENECA study respondents, sexual inactivity BNs, and dyspareunia BNs.

In the period between 15 May 2023 and 15 April 2024, questionnaires were sent electronically with up to two reminders to 38,657 women in the cohort. Among cervical cancer survivors, non-responders and those without e-Boks (the Danish national secure mail system) were contacted with a paper questionnaire between 1 October and 14 October 2024, retrieving responses from 409 additional women in the cohort. This provided 10,141 responses in total. In 39 respondents there was a discrepancy between self-reported and registered cervical cancer status, and these responses were omitted. Responses from 2002 out of 3746 cancer survivors and 7853 out of 34,409 reference women included at least one scale, corresponding to overall study response rates of 53.6% and 22.8% respectively. An additional 235 questionnaires from cervical cancer survivors diagnosed between 2020–2022 were included for prevalence analyses only because socioeconomic data required for BN analyses was not accessible. The participant inclusion flowchart is shown in Figure 1.

Danish national treatment guidelines for cervix cancer in the whole study period entailed that early stage disease was treated with surgery (cone, simple/radical hysterectomy or trachelectomy depending on stage and tumor diameter) with/without removal of lymph nodes. If the surgical specimen indicated high risk of recurrence, postoperative RCT was considered. In case of locally advanced disease, the standard treatment was RCT and brachytherapy.

Questionnaires developed and validated by the European Organization of Research and Treatment for Cancer group to assess quality of life in cancer patients (EORTC QLQ-C30) [19] and cervical cancer patients (EORTC QLQ-CX24) were included for analysis [20]. Disease, treatment, and demographic data were obtained from the Danish Gynecological Cancer Database (DGCD).

Respondents were included in the sexual activity BN model if they provided complete responses to all EORTC C30 and CX24 questions, except for the four conditional items that were only relevant to sexually active respondents (“pain during intercourse”, “sexual enjoyment”, “sexual/vaginal functioning”, “sexual worry”). For the dyspareunia BN, respondents who reported being sexually active and who had complete responses to all EORTC C30 and CX24 subscales were included. Due to the branching logic of the questionnaire, dyspareunia questions were administered only to women reporting sexual activity during the previous four weeks. Therefore, the dyspareunia analyses were restricted to sexually active respondents. Among the remaining participants, the degree of “sexual activity” was categorized using the EORTC response options—“a little”, “quite a bit” and “very much”. The “sexual worry” subscale was omitted from all models because of its strong correlation with the dyspareunia endpoint, which could confound results.

### 2.1. Data Preprocessing

Data was preprocessed following the EORTC scoring guidelines with minor modification to subscale definitions as required to fulfill the study objectives [21]. The “symptom experience” subscale, originally encompassing 13 questions, was divided into individual questions to allow greater detail in the analysis of mediating effects. Full description of the modified subscales used for analysis is included in Appendix A (Table A1).

Subscale scores were obtained by averaging responses within each subscale and normalizing to obtain a value between 0–100. Age and BMI at time of assessment were included for all respondents. The presence of tissue/organ involvement was categorized as “not involved” when the primary tumor was only affecting the cervix, and “tissue/organ involvement” if vagina, parametrium, bladder or rectum were involved.

### 2.2. Bayesian Network Modelling

Discrete BN structures were modelled for sexual activity and dyspareunia endpoints in both reference and cancer populations. Normalized EORTC subscales were discretized to obtain ordinal factors (Appendix A).

BN structure learning algorithms may be classified as constraint-based methods, utilizing statistical tests to identify conditional independence between factors, score-based methods, identifying a structure which optimizes an appropriate scoring function, or hybrid methods, which use a combination of constraint and score-based techniques.

In the reference population, BN structures were learnt using the Min–Max Hill Climbing (MMHC) hybrid structure learning algorithm [22]. Mutual information independence tests (alpha = 0.5) were applied to obtain an undirected acyclic graph. Arcs were directed using a greedy hill climbing algorithm to optimize the Bayesian Dirichlet equivalent (BDe) score with a uniform prior. The BDe score is a scoring method appropriate for discrete data [23]. The score may be computed with a uniform or custom prior [24].

Optimal imaginary sample size was computed by using the method of Steck for uniform priors iteratively until convergence on a stable value [25]. Bootstrapping was used to iterate the learning algorithm 1000 times, generating a measure of arc strength corresponding to the percentage of iterations in which a given arc was identified. Arcs with strength greater than 0.5 were included in the resulting model.

Network structures learnt from the reference population were used as prior distributions to learn models in the cancer population. BDe score with the Castelo & Siebe prior was used, applying arc strengths from the reference model as prior probability for arcs in the cancer population [24]. Arcs with strength below 0.5 were given equal probability. The Hill Climbing algorithm was then applied with cancer survivor cohort data, bootstrapped over 1000 iterations [26]. To separate the effects of age with treatment modality and tissue/organ involvement, the model was restricted from placing connecting arcs between age and radiotherapy or tissue/organ involvement, respectively.

The resulting models were subject to validation by domain-expert consensus, wherein arcs were selectively reversed to facilitate explainability. No arcs were added or removed in this process. Kendall rank correlation was computed between all pairs of connected nodes as a measure of positive or negative effect for model visualization. To validate our models identified through BN structure learning, Structural Equation Modelling (SEM) was used. SEMs are confirmatory models used to quantitatively assess hypothesized structural relationships using regression equations [27].

Structures were modelled using SEM by considering all nodes as observed variables and all arcs as regression equations and applying unweighted least squares mean- and variance-adjusted (ULSMVS) estimation to fit the models. Evaluation metrics were selected following recommended reporting practices [28] and included the chi-squared test, root mean square error of approximation (RMSEA), squared root mean residual (SRMR), comparative fit index (CFI), adjusted goodness-of-fit index (AGFI), and parsimony goodness-of-fit index (PNFI). Thresholds for acceptable and excellent model fit were taken from the literature [29] are included in Appendix B along with detailed descriptions of all evaluation metrics (Table A2). Regression coefficients with a standardized *p*-value less than 0.05 were considered significant arcs and included in the final models.

Modelling was performed in R (version 4.4.1) using the bnlearn (version 5.2.1) [30] and lavaan (version 0.7-2) [31] software packages.

## 3. Results

In 1945 out of 2237 (87%) cancer survivors and in 6838 out 7853 (87%) reference, information about sexual activity was available (Table 1). For the sexual activity BN models, complete responses to all EORTC C30 and CX24 questions, and clinical data where applicable, were available for 1694 out of 2237 (78%) cancer survivors and in 6706 out of 7853 (85%) references. Out of these, 996 cancer survivor and 4521 reference respondents were sexually active and had completed the vaginal and sexual functioning subscales and were included in the dyspareunia models.

The median age at time of assessment was 52 years for both the cancer and reference cohorts. Of 2237 cancer respondents, 1285 (56%) underwent surgery alone, 499 (22%) underwent RCT alone, and 453 (20%) received surgery and postoperative RCT.

Respondents were stratified by treatment modality; (1) reference group (*n* = 7853), (2) surgery alone (*n* = 1285), (3) RCT with/without prior surgery (*n* = 951). In the surgery cohort, 94% of respondents had disease only in the cervix and 1% had tissue/organ involvement (5% missing), with rates of 47% and 48% respectively in the RCT with/without surgery cohort (5% missing).

Table 1 shows prevalence rates of sexual activity and dyspareunia endpoints, as well as self-reported reasons for sexual inactivity in respondents who reported being “not at all” sexually active in the past four weeks.

**Figure 2:**
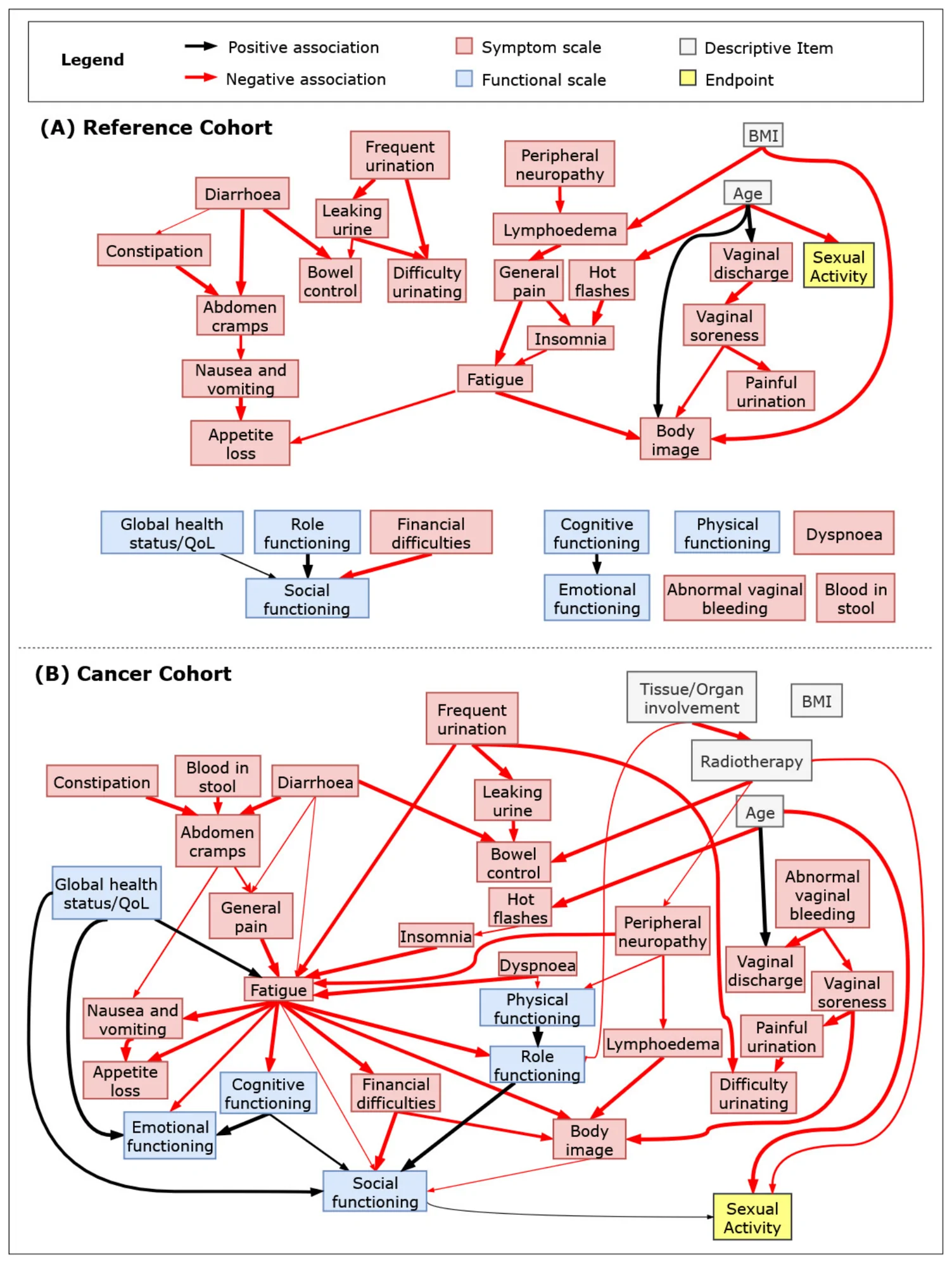
Bayesian network models for sexual activity: (**A**) reference cohort, (**B**) cancer survivor cohort.

BN structures for the sexual activity endpoint in reference and cancer populations are shown in Figure 2. If there is a negative association between nodes, corresponding to reduced function (negative correlation) or greater symptom severity (positive correlation), the arc is colored red. The strongest associations and the principal differences between the survivor and reference BNs are summarized below.

Table 2 shows a comparison of arc strength between the reference and cancer populations. All arcs with strength above the threshold of 0.5 in either structure were included, and the difference in arc strength was calculated by subtracting the cancer strength from reference strength for each arc. Thus, a positive difference indicates greater strength of association in the reference population and a negative difference indicates greater strength in the cancer population.

For example, Table 2 shows that the arc abnormal vaginal bleeding → vaginal discharge had a strength of 0.41 in the reference model, while the arc abnormal vaginal bleeding → vaginal soreness had a strength of 0.00. In the cancer model, these arcs had strengths of 0.97 and 0.85 respectively. Although both 0.41 and 0.00 are below the arc strength threshold of 0.5 for inclusion in the model, comparison of the arc strengths shows that the association with vaginal discharge is weakly present in the reference population while the association with vaginal soreness is unique to the cancer population.

**Figure 3:**
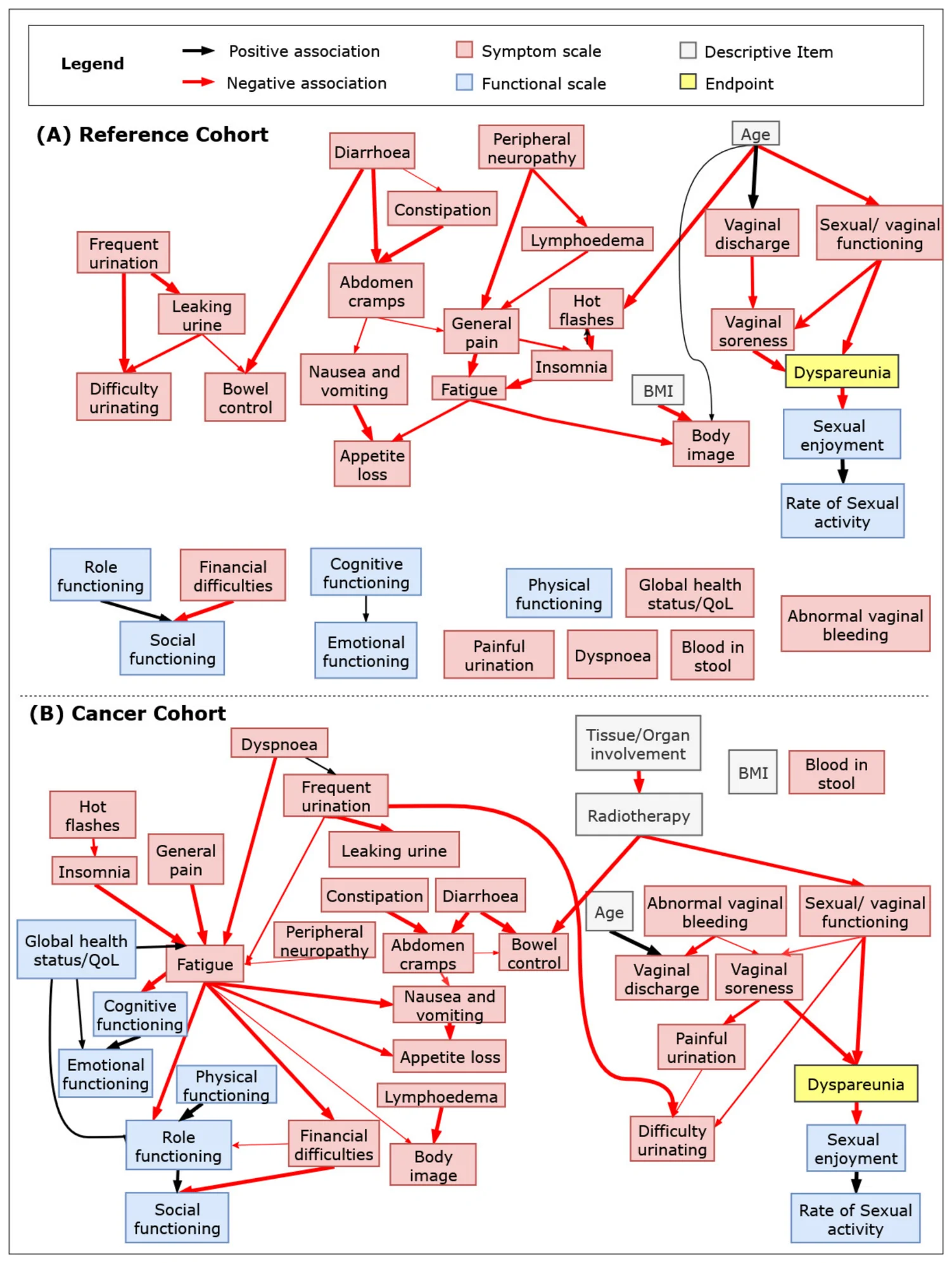
Bayesian network models for dyspareunia: (**A**) reference cohort, (**B**) cancer survivor cohort.

Figure 3 shows the BN structures for the dyspareunia endpoint, and Table 3 shows the analogous arc strength comparison.

Each model was validated through SEM, constructing an analogous network from the theorized BN structure to quantify goodness-of-fit. SEM evaluation metric results and regression coefficients are included in Appendix B.

All four SEM models had significant chi-squared test statistics (*p* < 0.0001), indicating poor model fit according to this metric. Because the chi-squared test is highly sensitive to large sample sizes, model fit was interpreted in conjunction with the remaining fit indices, all of which indicated acceptable (CFI, SRMR) to excellent (RMSEA, PNFI, AGFI) fit.

## 4. Discussion

### 4.1. Summary of Main Results

Sexual inactivity and dyspareunia were most prevalent among cervical cancer survivors who had undergone RCT, followed by those treated with surgery alone, and least prevalent among women without history of cancer. We constructed Bayesian network models of sexual activity and dyspareunia in reference and cancer survivor populations. Key pathways common to both models were age → sexual activity in the sexual activity model, and sexual/vaginal functioning → dyspareunia → sexual enjoyment → degree of sexual activity in the dyspareunia model. Treatment modality was strongly associated with bowel control and either sexual activity or sexual/vaginal functioning.

In the reference models, functional scales including emotional, cognitive, social, and role functioning exhibited few associations and appeared as isolated branches, while in the cancer models, they formed an integrated network showing prominent connections to fatigue and a direct association between social functioning and sexual activity. Comparison of arc strengths were used to quantify relative strength of association. Of factors relating to sexual function, strongest associations in the cancer survivor cohort compared to the reference population were seen for arcs between abnormal vaginal bleeding → vaginal soreness, vaginal soreness → painful urination, and sexual/vaginal functioning → body image.

### 4.2. Results in the Context of Published Literature

The results support previous findings that sexual inactivity and dyspareunia are more prevalent in cervical cancer patients treated with RCT than in those treated with surgery alone [32,33,34]. Vaginal morbidity (tightening, shortening, dryness, soreness) was the dominant indicator of dyspareunia in both reference and cancer survivor cohorts. While vaginal morbidity has been associated with both surgery and RCT [11], our results suggest that RCT was a stronger indicator of long-term vaginal morbidity and sexual inactivity due to cancer treatment.

Dyspareunia was reported by 44% of the sexually active cancer survivor cohort. This is in line with previous studies reporting rates of dyspareunia ranging from 16–55% in sexually active gynecological cancer patients [35,36,37]. Our results showed that 6%, 16%, and 21% of sexually inactive respondents in reference, surgery only, and RCT cohorts, respectively, reported dyspareunia as a reason for sexual inactivity. Thus, the reported figures likely underestimate the true prevalence of dyspareunia. As cancer patients are more frequently sexually inactive than reference women, the impact of underestimation is greater in cancer survivors compared to the reference population.

Previous studies have found fatigue to be significantly higher in cervical cancer survivors compared to the general population and have shown significant correlations with chronic fatigue and emotional functioning (depression, anxiety) as well as quality of life [38,39]. Our BN models provide additional support for this observation by highlighting the central role of fatigue within the cancer survivor cohort. In this model, fatigue emerged as a central node, connecting a wide range of both symptom (e.g., insomnia, pain, gastrointestinal symptoms) and functional domains (e.g., role functioning, social functioning, cognitive functioning). In contrast, the reference cohort model showed few meaningful associations between fatigue and other domains. Fatigue has a central role in the cancer model which underscores its clinical importance: addressing fatigue may not only improve energy levels but could also have wide-ranging benefits across several domains of survivorship.

BNs have been developed from PRO data to predict erectile dysfunction in prostate cancer patients [40]. In cervical cancer patients, BNs have been developed to predict survival outcomes [41] and to compare treatment modalities through meta-analysis [42]. To the best of our knowledge our study is the first application of BNs for explanatory modelling of sexual function in cervical cancer patients, as well as the first application of reference populations without history of cancer as structural priors for BN modeling in cancer populations.

### 4.3. Strengths and Weaknesses

The study included a large sample of cervical cancer survivors and reference women, which enabled modelling of complex associations and subgroup comparisons. Although the overall study response rates were 53.6% for cervical cancer survivors and 22.8% for reference women, completion of the sexual outcomes within participants who responded to at least one scale was high: 87% for sexual activity, and 97% for sexual/vaginal function in sexually active patients, for both reference and cancer cohorts. Further, comparison to population response rates from a study including 32,375 Danish women [43] showed comparable prevalence of sexual inactivity (28% in our study vs. 28.8% in the general population), indicating minimal effects of non-response bias [44]. The inclusion of a reference cohort enabled both direct comparisons with cancer survivors and the use of reference-derived structural priors during Bayesian network learning. Bayesian network structures were learned using a data-driven approach rather than being prespecified based on hypothesised relationships among variables. SEM analysis demonstrated validity of the models, showing acceptable overall fit. Although the chi-square test was significant, as expected for a large sample, and the SRMR (0.079) was close to the conventional threshold of 0.08 in the sexual activity cancer model, the remaining fit indices supported the adequacy of the model. The chi-squared test is highly sensitive to sample size and often rejects otherwise well-fitting models in large samples because even small deviations become statistically significant [45]. Therefore, when interpreted alongside other fit measures (RMSEA, SRMR, CFI, AGFI, PNFI), the chi-squared result does not undermine the validity of our models.

Limitations of this study included the inherent correlation between disease stage and treatment modality. In patients treated with RCT who reported greater levels of sexual dysfunction, it remains difficult to discern the extent to which prior tumor involvement, particularly in the vagina/parametrium, could have impacted these symptoms. Furthermore, the EORTC CX24 implicitly assumes sexual activity to mean penetrative intercourse, overlooking other forms of sexual intimacy. Reporting of sexual activity was limited to the past four weeks prior to assessment. This was seen to overestimate the rate of sexual inactivity as many respondents indicated infrequent sexual activity as a reason for selecting ’not at all’ sexually active. While dyspareunia was reported as a reason for sexual inactivity by 16–21% of sexually inactive cancer patients, EORTC CX24 responses were only recorded from sexually active respondents and could not be modelled in the full cohort. The resulting models may exclude respondents whose symptoms resulted in their sexual inactivity. Further, self-reported outcomes may be susceptible to reporting bias and subjective interpretation. Although the reference cohort was selected to have a similar age distribution, there is potential for residual confounding due to remaining differences in baseline characteristics. Additionally, directed arcs in the models cannot represent bidirectional relationships which may exist between factors, and causal interpretations should take this into consideration.

### 4.4. Implications for Practice and Further Research

Our results show a difference in the factors affecting sexual dysfunction for reference women and cancer survivors. Recommendations for sexual interventions should be tailored for the needs of cancer patients with the awareness that the factors affecting their sexual function differ from the general population. Whereas sexual activity in cancer survivors is associated with a complex pattern of general conditions such as fatigue, pain, insomnia and compromised social functioning, dyspareunia has less complex associations with general symptoms and is mainly linked with vaginal functioning. Management interventions in cancer patients and survivors should therefore focus both on general conditions as well as functioning of the sexual organs.

In the cancer survivor population, sexual inactivity was closely linked with reduced social functioning, which encompasses both social interactions and family life. Evidence suggests that psychosocial interventions—particularly couple counselling—can have a positive impact on sexual activity and body image in cancer survivors [46,47]. Strengthening access to and emphasis on psychosocial therapies may therefore play an important role in helping women manage sexual dysfunction after cancer treatment.

The strong association of fatigue with most functional domains, including social functioning, suggests interventions targeting fatigue may have a broad impact on overall quality of life and sexual function in cancer survivors. Physical exercise, particularly aerobic exercise, have been shown to improve cancer-related fatigue [48,49]. Cognitive Behavioral Therapy for insomnia has also shown significant benefits for cancer survivors through both in-person and online delivery [50,51]. The strong association between fatigue and functional domains is specific for cancer survivors and not seen in reference women. This indicates the need for a more holistic approach to cancer rehabilitation, including sexual rehabilitation. However, further longitudinal studies are needed to better understand the mechanisms linking fatigue with sexual dysfunction in cervical cancer survivors.

This study exhibits the value of BNs for creating explanatory models of cancer-related symptoms involving a wide range of risk factors. These models can be used to further expand our understanding of the complex interactions between symptoms affecting cancer survivors and inform development of comprehensive care practices to improve quality of life for cancer patients.

## 5. Conclusions

Sexual inactivity and dyspareunia were considerably more common among cervical cancer survivors than in the reference population, with the highest prevalence observed in women treated with RCT. Our explanatory Bayesian network models demonstrated that sexual dysfunction was linked not only to treatment modality and vaginal functioning but also by broader domains of health, particularly fatigue and its associations with functional status, quality of life, and social participation. Unlike the reference cohort, where associations were more limited, fatigue emerged as a central node in impaired functioning within the cancer cohort, underscoring its central role in survivorship.

The availability of a large reference cohort enabled direct comparison and strengthened the models, while the data-driven BN approach captured complex interdependencies without prespecifying risk factors. These findings point to the need for cancer-specific strategies in sexual rehabilitation that extend beyond local vaginal toxicity to address systemic late effects, especially fatigue, and their relationship with psychosocial and relational well-being. Integrating psychosocial interventions, fatigue management, and sexual health counselling into survivorship care pathways may help improve outcomes for cervical cancer survivors.

## Figures and Tables

**Table 1:** Prevalence of sexual outcomes in SENECA study.

	Reference Cohort		Surgery Only Cohort		Radio(chemo)therapy Cohort	
	#	%	#	%	#	%
Sexual Activity						
Not at all	2235	28%	378	29%	467	49%
A little	2719	35%	445	35%	257	27%
Quite a bit	1533	20%	224	17%	77	8%
Very much	351	4%	79	6%	18	2%
Missing	1015	13%	159	12%	132	14%
**Total**	**7853**		**1285**		**951**	
Dyspareunia *						
Not at all	3531	77%	447	60%	155	44%
A little	845	18%	218	29%	103	29%
Quite a bit	148	3%	42	6%	54	15%
Very much	75	2%	31	4%	34	10%
Missing	4	0%	10	1%	6	2%
**Total**	**4603**		**748**		**352**	
Characterisation of sexual inactivity **						
Infrequently active	N/A		200	53%	117	25%
Inactive before diagnosis *			52	14%	101	22%
Inactive since diagnosis *			121	32%	244	52%
Missing			5	1%	5	1%
**Total**			**378**		**467**	
Reason(s) for sexual inactivity ***						
No/Reduced desire	845	38%	195	52%	197	42%
Pain during sexual activity	126	6%	59	16%	99	21%
Bleeding during sexual activity	11	0%	6	2%	36	8%
Vagina felt tight	28	1%	32	8%	80	17%
Vagina felt short	10	0%	31	8%	93	20%
Vaginal dryness	125	6%	63	17%	86	18%
No partner	751	34%	134	35%	181	39%
Partner has sexual problems	324	14%	48	13%	41	9%
Do not know	233	10%	22	6%	35	7%
Other	294	13%	49	13%	56	12%

Notes: Surgery-only cohort includes cervical cancer patients treated with surgery and/or chemotherapy. Radio(chemo)therapy cohort includes patients treated with radiotherapy and/or chemotherapy and/or surgery. * Shown only to respondents who responded ‘a little’, ‘quite a bit’, or ‘very much’ sexually active in past four weeks. ** Shown only to respondents diagnosed with cervical cancer who responded ‘not at all’ sexually active in past four weeks. *** Shown only to respondents who responded ‘not at all’ sexually active. Respondents instructed to ‘select all that apply’.

**Table 2:** Comparison of arc strength in reference and cancer cohorts for sexual activity endpoint.

From	To	Ref.	Can.	Diff.
Abdomen cramps	General pain	0.04	**0.68**	–0.64
Abdomen cramps	Nausea and vomiting	**0.85**	**0.63**	0.22
**Abnormal vag. bleeding**	**Vagina discharge**	0.41	**0.97**	–0.56
**Abnormal vag. bleeding**	**Vagina soreness**	0.00	**0.85**	–0.85
Age	Body image	**0.94**	0.23	0.72
Age	Hot flashes	**1.00**	**1.00**	0.00
Age	**Sexual activity**	**1.00**	**1.00**	0.00
Age	**Vagina discharge**	**1.00**	**1.00**	0.00
BMI	Body image	**1.00**	0.06	0.95
BMI	Lymphoedema	**0.99**	0.31	0.68
Blood in stool	Abdomen cramps	0.00	**0.87**	–0.87
Body image	Social functioning	0.00	**0.53**	–0.53
Cognitive functioning	Emotional functioning	**0.90**	**0.97**	–0.07
Cognitive functioning	Social functioning	0.14	**0.72**	–0.58
Constipation	Abdomen cramps	**1.00**	**1.00**	0.00
Diarrhoea	Abdomen cramps	**1.00**	**1.00**	0.00
Diarrhoea	Bowel control	**1.00**	**1.00**	0.00
Diarrhoea	Constipation	**0.61**	0.35	0.26
Diarrhoea	Fatigue	0.00	**0.52**	–0.52
Diarrhoea	General pain	0.03	**0.56**	–0.54
Dyspnoea	Fatigue	0.00	**1.00**	–1.00
Dyspnoea	Physical functioning	0.00	**0.56**	–0.56
Fatigue	Appetite loss	**0.83**	**0.98**	–0.15
Fatigue	Body image	**0.99**	**0.95**	0.04
Fatigue	Cognitive functioning	0.00	**1.00**	–1.00
Fatigue	Emotional functioning	0.00	**0.82**	–0.82
Fatigue	Financial difficulties	0.08	**1.00**	–0.92
Fatigue	Nausea and vomiting	0.08	**1.00**	–0.92
Fatigue	Role functioning	0.00	**1.00**	–1.00
Fatigue	Social functioning	0.00	**0.54**	–0.54
Financial difficulties	Body image	0.00	**0.85**	–0.85
Financial difficulties	Social functioning	**1.00**	**1.00**	0.00
Frequent urination	Difficulty urinating	**1.00**	**0.99**	0.01
Frequent urination	Fatigue	0.05	**0.94**	–0.89
Frequent urination	Leaking urine	**1.00**	**1.00**	0.00
General pain	Fatigue	**1.00**	**1.00**	0.00
General pain	Insomnia	**0.93**	0.07	0.86
Global health status QoL	Emotional functioning	0.43	**0.96**	–0.53
Global health status QoL	Fatigue	0.00	**0.87**	–0.87
Global health status QoL	Social functioning	**0.59**	**0.87**	–0.28
Hot flashes	Insomnia	**1.00**	**0.61**	0.39
Insomnia	Fatigue	**1.00**	**1.00**	0.00
Leaking urine	Bowel control	**0.79**	**0.90**	–0.12
Leaking urine	Difficulty urinating	**0.98**	0.23	0.76
Lymphoedema	Body image	0.00	**0.99**	–0.99
Lymphoedema	General pain	**0.97**	0.06	0.91
Nausea and vomiting	Appetite loss	**1.00**	**1.00**	0.00
**Painful urination**	Difficulty urinating	0.41	**0.88**	–0.47
Peripheral neuropathy	Fatigue	0.08	**0.93**	–0.84
Peripheral neuropathy	General pain	**0.95**	0.28	0.68
Peripheral neuropathy	Lymphoedema	**0.90**	**0.74**	0.16
Peripheral neuropathy	Physical functioning	0.00	**0.61**	–0.61
Physical functioning	Role functioning	0.23	**1.00**	–0.77
Radiotherapy	Bowel control	NA	**1.00**	NA
Radiotherapy	Peripheral neuropathy	NA	**0.55**	NA
Radiotherapy	**Sexual activity**	NA	**0.71**	NA
Role functioning	Social functioning	**0.97**	**0.97**	0.00
Social functioning	**Sexual activity**	0.00	**0.51**	–0.51
Organ involvement	Radiotherapy	NA	**1.00**	NA
Organ involvement	Role functioning	NA	**0.58**	NA
**Vagina discharge**	**Vagina soreness**	**0.99**	**0.73**	0.26
**Vagina soreness**	Body image	**0.87**	**1.00**	–0.13
**Vagina soreness**	**Painful urination**	**0.93**	**0.99**	–0.06

Notes: Nodes related to vaginal functioning and sexual outcomes are bolded. Strengths above threshold of 0.5 which were considered for inclusion in final models are bolded. Responses related to cancer disease and treatment represented as NA in reference cohort. Difference was calculated as (Reference Strength − Cancer Strength).

**Table 3:** Comparison of arc strength in reference and cancer cohorts for dyspareunia endpoint.

From	To	Ref.	Can.	Diff.
Abdomen cramps	Nausea and vomiting	**0.61**	**0.70**	–0.09
**Abnormal vag. bleeding**	**Vagina discharge**	0.38	**0.85**	–0.47
**Abnormal vag. bleeding**	**Vagina soreness**	0.00	**0.55**	–0.55
Age	Body image	**0.55**	0.07	0.48
Age	Hot flashes	**1.00**	**1.00**	0.00
Age	**Sexual vaginal functioning**	**0.95**	0.00	0.95
Age	**Vagina discharge**	**1.00**	**1.00**	0.00
BMI	Body image	**1.00**	0.08	0.92
BMI	Lymphoedema	**0.99**	0.43	0.56
BMI	**Sexual vaginal functioning**	**0.50**	0.00	**0.50**
Cognitive functioning	Emotional functioning	**0.63**	**0.92**	–0.29
Constipation	Abdomen cramps	**1.00**	**1.00**	0.00
Diarrhoea	Abdomen cramps	**1.00**	**1.00**	0.00
Diarrhoea	Bowel control	**1.00**	**1.00**	0.00
Difficulty urinating	Painful urination	0.38	**0.55**	–0.18
**Dyspareunia**	**Sexual enjoyment**	**1.00**	**1.00**	0.00
Dyspnoea	Fatigue	0.00	**0.93**	–0.93
Dyspnoea	Frequent urination	0.00	**0.63**	–0.63
Fatigue	Appetite loss	0.21	**0.85**	–0.64
Fatigue	Body image	**0.85**	**0.54**	0.30
Fatigue	Cognitive functioning	0.00	**1.00**	–1.00
Fatigue	Financial difficulties	0.23	**0.97**	–0.75
Fatigue	Nausea and vomiting	0.17	**0.94**	–0.77
Fatigue	Role functioning	0.00	**0.93**	–0.93
Financial difficulties	Body image	0.00	**0.77**	–0.77
Financial difficulties	Role functioning	0.01	**0.52**	–0.52
Financial difficulties	Social functioning	**1.00**	**1.00**	0.01
Frequent urination	Difficulty urinating	**1.00**	**1.00**	0.00
Frequent urination	Fatigue	0.15	**0.65**	–0.50
Frequent urination	Leaking urine	**1.00**	**1.00**	0.00
General pain	Fatigue	**1.00**	**1.00**	0.00
General pain	Insomnia	**0.72**	0.13	0.59
Global health status QoL	Emotional functioning	0.13	**0.68**	–0.55
Global health status QoL	Fatigue	0.00	**0.77**	–0.77
Global health status QoL	Role functioning	0.00	**0.73**	–0.73
Hot flashes	Insomnia	**1.00**	**0.75**	0.25
Insomnia	Fatigue	**1.00**	**1.00**	0.00
Leaking urine	Bowel control	**0.62**	0.37	0.26
Leaking urine	Difficulty urinating	**0.82**	0.25	0.57
Lymphoedema	Body image	0.01	**0.99**	–0.98
Lymphoedema	General pain	**0.78**	0.02	0.75
Nausea and vomiting	Appetite loss	**1.00**	**1.00**	0.00
Organ involvement	Radiotherapy	NA	**1.00**	NA
Peripheral neuropathy	Fatigue	0.10	**0.52**	–0.41
Peripheral neuropathy	General pain	**0.96**	0.40	0.56
Peripheral neuropathy	Lymphoedema	**0.85**	0.36	0.49
Physical functioning	Role functioning	0.21	**0.99**	–0.78
Radiotherapy	Bowel control	NA	**1.00**	NA
Radiotherapy	**Sexual/vaginal function**	NA	**0.92**	NA
Role functioning	Social functioning	**0.87**	**0.86**	0.01
**Sexual enjoyment**	**Sexual activity**	**1.00**	**1.00**	0.00
**Sexual/vaginal function**	Body image	0.00	**0.66**	–0.66
**Sexual/vaginal function**	**Dyspareunia**	**1.00**	**1.00**	0.00
**Sexual/vaginal function**	**Vagina soreness**	**0.92**	**0.64**	0.28
**Vagina discharge**	**Vagina soreness**	**0.88**	0.07	0.81
**Vagina soreness**	**Dyspareunia**	**1.00**	**1.00**	0.00
**Vagina soreness**	Painful urination	0.06	**0.84**	–0.78

Notes: Nodes related to vaginal functioning and sexual outcomes are bolded. Strengths above threshold of 0.5 which were considered for inclusion in final models are bolded. Responses related to cancer disease and treatment represented as NA in reference cohort. Difference was calculated as (Reference Strength − Cancer Strength).

## Data Availability

The datasets presented in this article are not readily available due to data privacy. Requests to access the datasets should be directed to Peter Christensen.

## Abbreviations

The following abbreviations are used in this manuscript:

PRO	Patient Reported Outcome
RCT	Radio(chemo)therapy
QoL	Quality of Life
FIGO	International Federation of Gynaecology and Obstetrics
RT	Radiotherapy
BN	Bayesian Network
CRS	Civil Registration System
DGCD	Danish Gynecological Cancer Database
EORTC	European Organization of Research and Treatment for Cancer
MMHC	Min–Max Hill Climbing
BDe	Bayesian Dirichlet equivalent
SEM	Structural Equation Modelling
ULSMVS	Unweighted least squares mean and variance adjusted
RMSEA	Root mean square error of approximation
SRMR	Squared root mean residual
CFI	Comparative fit index
AGFI	Adjusted goodness-of-fit index
PNFI	Parsimony goodness-of-fit index

## Appendix A. Subscale Definition

Table A1 contains a complete account of subscales used to score the EORTC QLQ-C30 and CX24 questionnaires, including description of any modifications made to the EORTC recommended subscale definitions. Following EORTC scoring guidelines, linear transformations were applied to obtain subscale score [21].

Initial investigation of the data showed that the data was non-normal, and therefore not suitable for Gaussian BN modelling. Because many EORTC subscales are derived from a limited number of Likert-scale items (Table A1), the resulting transformed subscale scores have a relatively small number of ordinal, discrete values rather than behaving as continuous variables.

To obtain ordinal factors for BN model learning, the subscale score was discretized into three categories [0–32, 33–65, 66–100]. For single item scales, this is analogous to combining the categories of ‘Quite a bit’ and ‘Very much’ and was chosen to promote more even data distribution among categories as well as to account for subjective variations across patient interpretation of response categories.

Sensitivity analyses evaluated alternative discretisation strategies using two, three and four categories, and different cut-off values. Binary categorisation (“Not at all” vs. “A little” + “Quite a bit” + “Very much”) reduced the connectivity of BNs, primarily identifying age → sexual activity and age → dyspareunia or treatment modality → dyspareunia, and resulting in a greater number of isolated branches for all models.

Four-category discretization and alternative three-category methods (“Not at all” vs. “A little” + “Quite a bit” vs. “Very Much”) frequently resulted in insufficient observations to estimate conditional probabilities reliably because several response categories, particularly “Very Much,” were systematically underrepresented in the survey data. Additional tuning of the cut-off points for multi-item subscales had negligible effect on the resulting covariance matrix or BN structure. Based on these analyses, three categories [0–32, 33–65, 66–100] were selected for the final models.

**Table A1:** EORTC C30 and CX24 subscale modifications.

Subscale/Item	Number of Items	Item Range	QLQ-C30 and CX24 Item Numbers	Modified	Description of Modification
Global health status/QoL					
Global health status/QoL	2	6	29, 30	No	
Functional scales/items					
Physical functioning	5	3	1, 2, 3, 4, 5	No	
Role functioning	2	3	6, 7	No	
Emotional functioning	4	3	21, 22, 23, 24	No	
Cognitive functioning	2	3	20, 25	No	
Social functioning	2	3	26, 27	No	
Sexual activity	1	3	49	No	
Sexual enjoyment	1	3	54	No	
Symptom scales/items					
Fatigue	3	3	10, 12, 18	No	
Nausea and vomiting	2	3	14, 15	No	
Pain	3	3	9, 19, 39	Yes	Added item 39
Dyspnoea	1	3	8	No	
Insomnia	1	3	11	No	
Appetite loss	1	3	13	No	
Constipation	1	3	16	No	
Diarrhoea	1	3	17	No	
Financial difficulties	1	3	28	No	
Abdomen cramps	1	3	31	Yes	Removed from Symptom Experience
Bowel control	1	3	32	Yes	Removed from Symptom Experience
Blood in stools	1	3	33	Yes	Removed from Symptom Experience
Frequent urination	1	3	34	Yes	Removed from Symptom Experience
Painful urination	1	3	35	Yes	Removed from Symptom Experience
Leaking urination	1	3	36	Yes	Removed from Symptom Experience
Difficulty urinating	1	3	37	Yes	Removed from Symptom Experience
Vagina soreness	1	3	41	Yes	Removed from Symptom Experience
Vagina discharge	1	3	42	Yes	Removed from Symptom Experience
Abnormal vaginal bleeding	1	3	43	Yes	Removed from Symptom Experience
Body image	3	3	45, 46, 47	No	
Sexual/Vaginal functioning	3	3	50, 51, 52	Yes	Removed item 53
					Removed from Sexual/Vaginal
Dyspareunia	1	3	53	Yes	functioning
Lymphoedema	1	3	38	No	
Peripheral neuropathy	1	3	40	No	
Menopausal symptoms	1	3	44	No	
Sexual worry	1	3	48	No	

## Appendix B. Structural Equation Modelling

### Appendix B.1. Evaluation Metrics

Table A2 contains metrics used to evaluate the SEM models. Criteria for acceptable and excellent fit were derived from the literature [28]. Metrics were chosen following recommended reporting practices [29] to include a combination of absolute, incremental, and parsimony fit indices.

Absolute fit indices compare the model to the sample data. This includes the chi-square value, which assess the null hypothesis that the model is a true representation of the sample covariance matrices. An insignificant result (*p* > 0.05) indicates good model fit. This metric is sensitive to sample-size and non-normality. Root Mean Square Error of Approximation (RMSEA) is an absolute metric that assesses error between the modeled and sampled covariance matrices. Error less than 0.05 indicates excellent fit. A confidence interval can be calculated for this metric, strengthening its reliability. Similarly to the chi-square statistic, an insignificant result (*p* > 0.05) indicates good model fit. The Adjusted Goodness-of-fit index (AGFI) evaluates the proportion of variance between the modelled and sampled covariance matrices. This metric is adjusted based on degrees of freedom in the model, and is sensitive to sample size. A value of 1 indicates perfect fit. The Standardized Root Mean Square Residual (SRMR) evaluates the difference between the residuals of the modelled and sampled covariance matrixes and is standardized between 0 and 1, with values below 0.05 considered excellent fit.

Incremental fit indices compare the hypothesized model to a baseline model. The Comparative Fit Index (CFI) utilizes a “worst-case” baseline model by assuming that all latent variables are uncorrelated. By comparing the computed model to this baseline model, a measure of fit is obtained. This index is particularly robust to sample size variations. A value of 1 indicates perfect fit.

Parsimony fit indices aim to prioritize simplicity alongside model fit. The Parsimony Normed Fit Index (PNFI) is a product of the Parsimony Ratio and Normed Fit Index which accounts for complexity of the model in addition to goodness-of-fit compared to a baseline model. Values greater than 0.5 are considered acceptable fit.

**Table A2:** Evaluation metrics for SEM models.

Metric	Thresholds		Results			
	Acceptable Fit	Excellent Fit	Sexual Activity		Dyspareunia	
			Reference Cohort	Cancer Cohort	Reference Cohort	Cancer Cohort
Chi Squared scaled	p>0.05	–	5241	2634	3323	1848
			(p<0.001)	(p<0.001)	(p<0.001)	(p<0.001)
RMSEA robust	<0.08, p>0.05	<0.05, p>0.05	0.018 (p=1.000)	0.027 (p=1.000)	0.017 (p=1.000)	0.024 (p=1.000)
SRMR	<0.08	<0.05	0.06	0.079	0.069	0.068
CFI robust	>0.9	>0.95	0.907	0.969	0.902	0.914
AGFI	>0.9	>0.95	0.975	0.958	0.975	0.956
PNFI scaled	>0.5	–	0.614	0.656	0.624	0.581

Notes: Robust and scaled metrics reported if available. Abbreviations: RMSEA—Root Mean Square Error of Approximation; SRMR—Standardized Root Mean Residual; CFI—Comparative Fit Index; AGFI—Adjusted Goodness-of-Fit Index; PNFI—Parsimony Normed Fit Index.

### Appendix B.2. Regression Coefficients

Table A3, Table A4, Table A5 and Table A6 show scaled regression coefficient estimates for the sexual activity reference cohort, sexual activity cancer cohort, dyspareunia reference cohort, and dyspareunia cancer cohort SEMs respectively. Regressions with *p*-value less than 0.05 were considered significant and included in the final BN models.

**Table A3:** Sexual activity reference cohort SEM model scaled regression coefficient estimates.

To	From	Point Estimate (95% CI)	SE	*p*-Value
Abdomen cramps	Constipation	0.178 (0.153; 0.203)	0.013	<0.001
Abdomen cramps	Diarrhoea	0.183 (0.151; 0.215)	0.016	<0.001
Abdomen cramps	Nausea and vomiting	0.664 (0.590; 0.738)	0.038	<0.001
Appetite loss	Nausea and vomiting	0.706 (0.633; 0.779)	0.037	<0.001
Body image	Age	−0.042 (−0.055; −0.029)	0.007	<0.001
Body image	BMI	0.091 (0.075; 0.106)	0.008	<0.001
Body image	Fatigue	0.637 (0.591; 0.682)	0.023	<0.001
Body image	Vagina soreness	0.280 (0.248; 0.312)	0.017	<0.001
Bowel control	Diarrhoea	0.250 (0.224; 0.277)	0.014	<0.001
Bowel control	Leaking urine	0.112 (0.092; 0.133)	0.010	<0.001
Constipation	Diarrhoea	0.302 (0.266; 0.337)	0.018	<0.001
Difficulty urinating	Frequent urination	0.310 (0.282; 0.339)	0.015	<0.001
Difficulty urinating	Leaking urine	0.058 (0.030; 0.087)	0.015	<0.001
Emotional functioning	Cognitive functioning	0.328 (0.257; 0.400)	0.036	<0.001
Emotional functioning	Global health status QoL	0.340 (0.264; 0.416)	0.039	<0.001
Fatigue	Appetite loss	2.144 (1.915; 2.374)	0.117	<0.001
Fatigue	General pain	0.179 (0.143; 0.216)	0.019	<0.001
Financial difficulties	General pain	0.554 (0.502; 0.606)	0.027	<0.001
General pain	Lymphoedema	0.143 (0.121; 0.166)	0.012	<0.001
General pain	Peripheral neuropathy	0.009 (−0.022; 0.041)	0.016	0.560
General pain	Insomnia	1.064 (0.987; 1.140)	0.039	<0.001
Hot flashes	Age	0.124 (0.109; 0.138)	0.007	<0.001
Hot flashes	Body image	0.607 (0.538; 0.675)	0.035	<0.001
Insomnia	Hot flashes	0.782 (0.684; 0.880)	0.050	<0.001
Insomnia	Fatigue	0.444 (0.412; 0.476)	0.016	<0.001
Leaking urine	Frequent urination	0.408 (0.380; 0.437)	0.014	<0.001
Lymphoedema	BMI	0.170 (0.152; 0.189)	0.010	<0.001
Lymphoedema	Peripheral neuropathy	0.385 (0.349; 0.420)	0.018	<0.001
Painful urination	Vagina soreness	0.116 (0.093; 0.139)	0.012	<0.001
Sexual activity	Age	−0.164 (−0.181; −0.148)	0.008	<0.001
Social functioning	Financial difficulties	−0.175 (−0.205; −0.146)	0.015	<0.001
Social functioning	Global health status QoL	0.423 (0.323; 0.522)	0.051	<0.001
Social functioning	Role functioning	0.158 (0.100; 0.216)	0.030	<0.001
Vagina discharge	Age	−0.243 (−0.254; −0.231)	0.006	<0.001
Vagina soreness	Vagina discharge	0.098 (0.072; 0.125)	0.014	<0.001

Abbreviations: CI—Confidence Interval; SE—Standard Error; SEM—Structural Equation Model.

**Table A4:** Sexual activity cancer cohort SEM model scaled regression coefficient estimates.

To	From	Point Estimate (95% CI)	SE	*p*-Value
Abdomen cramps	Blood in stool	2.840 (2.072; 3.607)	0.392	<0.001
Abdomen cramps	Constipation	0.140 (0.052; 0.227)	0.045	0.002
Appetite loss	Fatigue	0.230 (0.182; 0.278)	0.024	<0.001
Appetite loss	Nausea and vomiting	0.365 (0.284; 0.447)	0.042	<0.001
Body image	Fatigue	0.392 (0.312; 0.472)	0.041	<0.001
Body image	Lymphoedema	0.152 (0.109; 0.194)	0.022	<0.001
Body image	Vagina soreness	0.241 (0.188; 0.293)	0.027	<0.001
Body image	Financial difficulties	0.121 (0.053; 0.189)	0.035	<0.001
Bowel control	Diarrhoea	0.407 (0.356; 0.459)	0.026	<0.001
Bowel control	Leaking urine	0.212 (0.171; 0.254)	0.021	<0.001
Bowel control	Radiotherapy	0.458 (0.370; 0.546)	0.045	<0.001
Cognitive functioning	Fatigue	−0.286 (−0.332; −0.240)	0.023	<0.001
Diarrhoea	Abdomen cramps	0.567 (0.507; 0.627)	0.031	<0.001
Difficulty urinating	Frequent urination	0.314 (0.270; 0.359)	0.023	<0.001
Difficulty urinating	Painful urination	0.686 (0.538; 0.834)	0.076	<0.001
Emotional functioning	Cognitive functioning	0.221 (0.144; 0.297)	0.039	<0.001
Emotional functioning	Global health status QoL	0.157 (0.053; 0.262)	0.053	0.003
Emotional functioning	Fatigue	−0.049 (−0.083; −0.016)	0.017	0.004
Fatigue	General pain	0.422 (0.381; 0.464)	0.021	<0.001
Fatigue	Global health status QoL	−2.770 (−3.469; −2.071)	0.357	<0.001
Fatigue	Diarrhoea	0.155 (0.116; 0.195)	0.020	<0.001
Fatigue	Dyspnoea	−0.331 (−0.543; −0.120)	0.108	0.002
Financial difficulties	Fatigue	0.552 (0.491; 0.613)	0.031	<0.001
Frequent urination	Fatigue	0.633 (0.573; 0.693)	0.031	<0.001
General pain	Abdomen cramps	0.458 (0.394; 0.521)	0.032	<0.001
General pain	Diarrhoea	0.136 (0.079; 0.193)	0.029	<0.001
Insomnia	Hot flashes	0.095 (0.050; 0.140)	0.023	<0.001
Insomnia	Fatigue	0.653 (0.588; 0.718)	0.033	<0.001
Leaking urine	Frequent urination	0.619 (0.568; 0.670)	0.026	<0.001
Lymphoedema	Peripheral neuropathy	0.818 (0.704; 0.932)	0.058	<0.001
Nausea and vomiting	Abdomen cramps	0.123 (0.076; 0.170)	0.024	<0.001
Nausea and vomiting	Fatigue	0.240 (0.190; 0.290)	0.025	<0.001
Painful urination	Vagina soreness	0.578 (0.473; 0.682)	0.053	<0.001
Peripheral neuropathy	Radiotherapy	0.261 (0.176; 0.345)	0.043	<0.001
Peripheral neuropathy	Fatigue	0.566 (0.509; 0.623)	0.029	<0.001
Physical functioning	Dyspnoea	−0.096 (−0.131; −0.061)	0.018	<0.001
Physical functioning	Peripheral neuropathy	−0.037 (−0.058; −0.016)	0.011	0.001
Radiotherapy	Organ involvement	0.749 (0.698; 0.800)	0.026	<0.001
Role functioning	Fatigue	−0.268 (−0.316; −0.220)	0.024	<0.001
Role functioning	Physical functioning	0.932 (0.544; 1.319)	0.198	<0.001
Role functioning	Organ involvement	−0.138 (−0.213; −0.064)	0.038	<0.001
Sexual activity	Age	−0.189 (−0.221; −0.158)	0.016	<0.001
Sexual activity	Radiotherapy	−0.257 (−0.339; −0.175)	0.042	<0.001
Sexual activity	Social functioning	0.677 (0.529; 0.825)	0.075	<0.001
Social functioning	Body image	−0.039 (−0.074; −0.005)	0.018	0.026
Social functioning	Role functioning	0.237 (0.153; 0.321)	0.043	<0.001
Social functioning	Cognitive functioning	0.169 (0.082; 0.256)	0.044	<0.001
Social functioning	Fatigue	−0.133 (−0.194; −0.072)	0.031	<0.001
Social functioning	Financial difficulties	−0.103 (−0.154; −0.052)	0.026	<0.001
Vagina discharge	Abnormal vaginal bleeding	2.255 (1.440; 3.071)	0.416	<0.001
Vagina discharge	Age	−0.166 (−0.191; −0.140)	0.013	<0.001
Vagina soreness	Abnormal vaginal bleeding	4.019 (2.613; 5.425)	0.717	<0.001
Vagina soreness	Vagina discharge	0.080 (−0.079; 0.238)	0.081	0.323

Abbreviations: CI—Confidence Interval; SE—Standard Error; SEM—Structural Equation Model.

**Table A5:** Dyspareunia reference cohort SEM model scaled regression coefficient estimates.

To	From	Point Estimate (95% CI)	SE	*p*-Value
Abdomen cramps	Constipation	0.246 (0.218; 0.274)	0.014	<0.001
Abdomen cramps	Diarrhoea	0.307 (0.276; 0.338)	0.016	<0.001
Appetite loss	Nausea and vomiting	0.678 (0.597; 0.758)	0.041	<0.001
Body image	Fatigue	0.599 (0.548; 0.650)	0.026	<0.001
Body image	BMI	0.106 (0.085; 0.126)	0.011	<0.001
Body image	Age	−0.042 (−0.058; −0.026)	0.008	<0.001
Bowel control	Diarrhoea	0.215 (0.185; 0.244)	0.015	<0.001
Bowel control	Leaking urine	0.120 (0.092; 0.148)	0.014	<0.001
Difficulty urinating	Frequent urination	0.468 (0.403; 0.534)	0.033	<0.001
Difficulty urinating	Leaking urine	0.288 (0.224; 0.353)	0.033	<0.001
Dyspareunia	Vagina soreness	0.404 (0.354; 0.454)	0.026	<0.001
Dyspareunia	Sexual vaginal functioning	0.427 (0.390; 0.465)	0.019	<0.001
Emotional functioning	Cognitive functioning	0.531 (0.418; 0.645)	0.058	<0.001
Fatigue	Insomnia	0.339 (0.183; 0.495)	0.080	<0.001
Fatigue	General pain	0.757 (0.610; 0.903)	0.075	<0.001
Frequent urination	Fatigue	0.566 (0.498; 0.635)	0.035	<0.001
General pain	Lymphoedema	0.677 (0.580; 0.774)	0.049	<0.001
General pain	Peripheral neuropathy	0.956 (0.806; 1.105)	0.076	<0.001
General pain	Abdomen cramps	0.768 (0.692; 0.844)	0.039	<0.001
Hot flashes	Age	0.117 (0.100; 0.134)	0.009	<0.001
Insomnia	Hot flashes	0.161 (0.125; 0.196)	0.018	<0.001
Insomnia	General pain	0.803 (0.731; 0.875)	0.037	<0.001
Leaking urine	Frequent urination	0.631 (0.543; 0.720)	0.045	<0.001
Lymphoedema	Peripheral neuropathy	0.854 (0.709; 0.999)	0.074	<0.001
Lymphoedema	BMI	−4.446 (−11.738; 2.845)	3.720	0.232
Nausea and vomiting	Abdomen cramps	0.285 (0.252; 0.318)	0.017	<0.001
Sexual activity	Sexual enjoyment	0.230 (0.202; 0.258)	0.014	<0.001
Sexual enjoyment	Dyspareunia	−0.274 (−0.312; −0.237)	0.019	<0.001
Sexual vaginal functioning	Age	0.097 (0.080; 0.113)	0.008	<0.001
Social functioning	Role functioning	0.309 (0.231; 0.388)	0.040	<0.001
Social functioning	Financial difficulties	−0.230 (−0.278; −0.181)	0.025	<0.001
Vagina discharge	Age	−0.235 (−0.250; −0.220)	0.008	<0.001
Vagina soreness	Vagina discharge	0.112 (0.082; 0.142)	0.015	<0.001
Vagina soreness	Sexual vaginal functioning	0.311 (0.277; 0.344)	0.017	<0.001

Abbreviations: CI—Confidence Interval; SE—Standard Error; SEM—Structural Equation Model.

**Table A6:** Dyspareunia cancer cohort SEM model scaled regression coefficient estimates.

To	From	Point Estimate (95% CI)	SE	*p*-Value
Abdomen cramps	Constipation	0.166 (0.090; 0.242)	0.039	<0.001
Abdomen cramps	Diarrhoea	1.019 (0.837; 1.201)	0.093	<0.001
Appetite loss	Fatigue	0.212 (0.159; 0.265)	0.027	<0.001
Appetite loss	Nausea and vomiting	0.294 (0.184; 0.405)	0.056	<0.001
Body image	Fatigue	0.590 (0.504; 0.675)	0.044	<0.001
Body image	Financial difficulties	−0.016 (−0.090; 0.058)	0.038	0.679
Body image	Sexual vaginal functioning	0.197 (0.148; 0.246)	0.025	<0.001
Body image	Lymphoedema	0.135 (0.096; 0.174)	0.020	<0.001
Bowel control	Diarrhoea	0.434 (0.373; 0.495)	0.031	<0.001
Bowel control	Radiotherapy	0.455 (0.360; 0.550)	0.048	<0.001
Cognitive functioning	Fatigue	−0.260 (−0.320; −0.200)	0.031	<0.001
Diarrhoea	Fatigue	0.486 (0.405; 0.567)	0.041	<0.001
Difficulty urinating	Frequent urination	0.878 (0.695; 1.060)	0.093	<0.001
Dyspareunia	Vagina soreness	0.456 (0.388; 0.524)	0.035	<0.001
Dyspareunia	Sexual vaginal functioning	0.469 (0.409; 0.530)	0.031	<0.001
Emotional functioning	Cognitive functioning	0.192 (0.097; 0.287)	0.048	<0.001
Emotional functioning	Role functioning	0.172 (0.046; 0.299)	0.065	0.008
Fatigue	Insomnia	0.144 (0.089; 0.198)	0.028	<0.001
Fatigue	Dyspnoea	0.908 (0.747; 1.069)	0.082	<0.001
Fatigue	Frequent urination	0.105 (0.049; 0.161)	0.028	<0.001
Fatigue	Peripheral neuropathy	0.203 (0.149; 0.258)	0.028	<0.001
Fatigue	General pain	0.455 (0.411; 0.500)	0.023	<0.001
Financial difficulties	Fatigue	0.486 (0.407; 0.565)	0.040	<0.001
Frequent urination	Dyspnoea	0.737 (0.576; 0.898)	0.082	<0.001
Global health status QoL	Fatigue	−0.139 (−0.181; −0.096)	0.021	<0.001
Hot flashes	Age	0.020 (−0.007; 0.048)	0.014	0.139
Hot flashes	Body image	0.553 (0.433; 0.674)	0.061	<0.001
Insomnia	Hot flashes	1.491 (1.165; 1.818)	0.166	<0.001
Leaking urine	Frequent urination	0.507 (0.446; 0.568)	0.031	<0.001
Nausea and vomiting	Fatigue	0.172 (0.106; 0.237)	0.034	<0.001
Nausea and vomiting	Abdomen cramps	0.175 (0.086; 0.264)	0.045	<0.001
Painful urination	Difficulty urinating	0.096 (0.058; 0.133)	0.019	<0.001
Painful urination	Vagina soreness	0.288 (0.197; 0.378)	0.046	<0.001
Radiotherapy	Organ involvement	1.219 (1.105; 1.333)	0.058	<0.001
Role functioning	Physical functioning	2.379 (1.382; 3.377)	0.509	<0.001
Role functioning	Fatigue	−0.178 (−0.235; −0.121)	0.029	<0.001
Role functioning	Financial difficulties	−0.098 (−0.167; −0.029)	0.035	0.006
Sexual activity	Sexual enjoyment	0.373 (0.281; 0.466)	0.047	<0.001
Sexual enjoyment	Dyspareunia	−0.464 (−0.585; −0.343)	0.062	<0.001
Sexual vaginal functioning	Radiotherapy	1.093 (0.729; 1.456)	0.185	<0.001
Social functioning	Role functioning	0.535 (0.343; 0.728)	0.098	<0.001
Social functioning	Financial difficulties	−0.202 (−0.275; −0.130)	0.037	<0.001
Vagina discharge	Abnormal vaginal bleeding	1.381 (0.836; 1.926)	0.278	<0.001
Vagina discharge	Age	−0.177 (−0.212; −0.142)	0.018	<0.001
Vagina soreness	Abnormal vaginal bleeding	1.092 (0.739; 1.446)	0.180	<0.001
Vagina soreness	Sexual vaginal functioning	0.959 (0.781; 1.137)	0.091	<0.001

Abbreviations: CI—Confidence Interval; SE—Standard Error; SEM—Structural Equation Model.
